# Group Rumination: Social Interactions Around Music in People with Depression

**DOI:** 10.3389/fpsyg.2017.00490

**Published:** 2017-04-04

**Authors:** Sandra Garrido, Tuomas Eerola, Katrina McFerran

**Affiliations:** ^1^MARCS Institute for Brain, Behaviour and Development, Western Sydney UniversityMilperra, NSW, Australia; ^2^Department of Music, Durham UniversityDurham, UK; ^3^University of MelbourneMelbourne, VIC, Australia

**Keywords:** rumination, depression, music listening, social feedback, coping style

## Abstract

One of the most important roles that music serves in human society is the promotion of social relationships and group cohesion. In general, emotional experiences tend to be amplified in group settings through processes of social feedback. However, previous research has established that listening to sad music can intensify negative emotions in people with tendencies to rumination and depression. This study therefore investigated the phenomenon of ruminating with music, and the question of whether listening to sad music in group settings provides social benefits for emotionally vulnerable listeners, or whether it further exaggerates depressive tendencies. Participants recruited via online depression groups and mental health websites were surveyed as to music listening habits. Results revealed that people with depression were more likely to engage in “group rumination” using music, and that this behavior could be partially explained by a general tendency to ruminate using music. Both affective states and coping styles were found to be related to the affective outcomes of group interactions around music. These findings go some way toward clarifying the situations in which group interactions around music are able to provide important social benefits for those involved, and situations in which negative emotions can be amplified by the group context.

## Introduction

Emotions serve adaptive social functions, including assisting individuals to create and sustain relationships, and to establish or preserve their place within the social hierarchies of a group (Fischer and Greitmeyer, 2006). Positive emotions particularly, serve so as to cement social bonds within a group. However, demonstrations of negative emotions like crying, tend to signal weakness and the need for social support (Balsters et al., 2013). Such displays of vulnerability can diminish one's social standing within a group. However, people in close relationships are more likely to respond to displays of negative emotions with offers of support. Thus people tend to cry more often in the presence of intimates than in public situations (Vingerhoets and Becht, 1997). Higher mood improvements from crying are reported where such support is received (Bylsma et al., 2011), suggesting that the social context in which crying occurs has an influence on the psychological effect experienced.

In much the same way that expressions of emotion strengthen social bonds, music also bolsters social relationships throughout the lifespan. In the early years, musical rituals consolidate parental-infant bonds (Trehub and Trainor, 1998). Music also creates and strengthens interpersonal relations between peers among adolescents and young adults because of the signals that one's taste in music gives about shared values (Boer et al., 2011). Music is thus used as an “identity badge” (Frith, 1981; Tarrant et al., 2001), and in the formation of friendships (Selfhout et al., 2009).

The act of creating or enjoying music alongside other individuals similarly has an impact on social relationships. Evidence suggests that when members of a group synchronize to a beat, this influences their behavior toward both individuals within the group and those perceived as being outside it. Kirschner and Tomasello (2010), for example, found that 4-year olds who synchronized movements and playing of percussion instruments in a group activity were more likely to exhibit cooperative and helpful behaviors afterwards compared to a control group that did no musical activities. Similar effects on prosocial behaviors and group affiliations have been found in both children and adults after activities involving coordinating their movements to a simple rhythm (Hove and Risen, 2009; Cirelli et al., 2014).

Since both the sharing of emotions and the sharing of music provide similar social benefits, it could be presumed that situations in which individuals share *emotional* experiences with music with others, are an especially powerful form of social bonding. In fact, evidence suggests that emotional experiences with music are amplified in group-settings. Emotional contagion is believed to take place through processes of unconscious motor mimicry which cause individuals to experience parallel emotions to those perceived in other people (Hatfield et al., 2011). Social facilitation theory also suggests that arousal levels increase in the presence of others (Zajonc and Sales, 1966). Thus, the most powerful emotional experiences in response to music often occur at live events where other people are present (Lamont, 2009), a phenomenon also observed in movie theaters (Coplan, 2006). This emotional amplification is believed to take place through processes of social feedback (Egermann et al., 2013).

Given the role of crying in gaining social support, the question thus arises as to whether the sharing of *sad music* in group contexts holds similar benefits for members of the group. The potential for emotional responses to be intensified in group settings is of particular interest when considering music that evokes negative emotions. In fact, entire musical subcultures such as “goth” or “emo” are focused on music with dark and depressing themes. Such music has taken the blame in cases of suicide, such as the double suicide in 2007 of two teenage girls in Australia who had made multiple posts on social media about music that echoed their negative views of the world (Young et al., 2014).

In fact, previous research has demonstrated that listening to music that is perceived as sad can intensify depression in listeners with ruminative tendencies (Garrido and Schubert, 2015a,b), and that people with depressive tendencies may struggle to regulate their emotional responses in musical contexts (McFerran, 2016). Since group settings and social feedback can intensify the emotions experienced in response to music, the possibility exists that group interactions around sad music could have an even more dangerous outcome for people with tendencies to depression. Rose (2002) described the extensive discussion and revisiting of problems among friends as “co-rumination,” and the term has also been applied to group interactions about music that focus on personal problems and negative thoughts (Miranda et al., 2012). The combination of music that focuses on depressing themes along with excessive listening and discussion of such music in a kind of “group rumination” may be a particularly detrimental mix for vulnerable individuals.

On the other hand, it may be possible that the sharing of emotions via music listening in group settings provides depressed individuals with needed social support and enhances their sense of connection with other individuals. In general, “reflection” as distinct from rumination, provides a useful psychological tool for processing negative emotions (Trapnell and Campbell, 1999), and it could be that similar processes are also occurring in group interactions around music. It may be that individuals with adaptive coping strategies such as reflectiveness or the seeking of social support, may use group interactions around music in positive ways so as to gain important support or to help them process negative emotions. Thus the questions of how social settings influence the affective outcomes of listening to music and how this is related to coping style, are important ones to consider in exploring the relationship between music and mental health and wellbeing. The aims of the current study are, therefore:

1. To investigate the self-reported mood effects of listening to sad music in group settings.
2. To investigate the influence of rumination, depression, and coping style on the mood effects reported.

## Hypotheses

*H1: People with depression will tend to ruminate with music during general listening and engage in “group rumination” more than others*.
*H2: The relationship between depression and group rumination will be mediated by a general tendency to ruminate using music*.
*H3: People with adaptive coping styles will experience positive mood effects from listening to their self-selected music when sad*.
*H4: People with adaptive coping styles will be more likely to use music in positive ways in group settings*.

## Methods

### Participants

After obtaining ethics approval from the relevant boards at both the University of Melbourne and Durham University, 697 participants were recruited via advertisements on mental health and depression websites in Australia, the U.K. and the U.S, including 196 males and 501 females. Similar gender imbalances are often found in studies relating to mental health issues such as depression (see for e.g., Lindner et al., 2016), and likely reflect the higher incidence of depression in females than males (Freeman and Freeman, 2013). Participants ranged in age from 16 to 74 years of age (*m* = 24.9, *SD* = 11) and were mostly from the U.S. (72.3%), Australia (10.3%), and the U.K. (5.2%), with 12.2% being from a number of other countries in South America, Africa, Asia, and Europe.

### Procedure

Participants completed an online survey in their own time in which they were asked a number of questions relating to their ways of using music, types of musical engagement and the effect of music listening (see Appendix). They were also asked to nominate a song that they would listen to when feeling sad.

### Measures

Participants completed the short form of the Depression-Anxiety-Stress Scale (DASS-21; Henry and Crawford, 2005). The DASS-21 is a 21-item questionnaire in which participants indicate the presence of symptoms of depression, anxiety and stress in the past week. Items are scored on a scale of 0 (*did not apply to me at all over the last week*) to 3 (*applied to me very much or most of the time over the past week*). In the current study Cronbach's alpha scores were 0.93 (Depression), 0.88 (Stress), and 0.87 (Anxiety).

In addition to measuring current symptoms, general tendencies to depression were measured using the Rumination Reflection Questionnaire (RRQ; Trapnell and Campbell, 1999). The RRQ was designed to distinguish between a maladaptive focus on negative thoughts (rumination) and healthier tendencies to self-reflection (reflectiveness). Rumination generally, is highly predictive of depression (Nolen-Hoeksema and Morrow, 1993). Since the items in the rumination subscale measure long-term coping styles, the scale is indicative of tendencies to depression rather than the presence of a depressive episode. In the current study reliability estimates were 0.91 and 0.88 for the rumination and reflectiveness subscales respectively (Cronbach's alpha).

The Healthy Uses of Music Scale (HUMS) (Saarikallio et al., 2015) was also included, a scale developed to assess musical engagement as an indicator of wellbeing. The HUMS consists of 13 items of which five measure healthy and eight measure unhealthy dimensions of musical engagement. Reliability estimates in the current study were 0.77 for the healthy subscale, and 0.83 for the unhealthy subscale.

Participants also completed 23 items designed by the authors to examine various aspects of music listening both in solitary and in group situations (see Appendix). These items included 3 items relating to some of the reasons that people frequently give for listening to sad music (Garrido and Schubert, 2013), and 3 items relating to the emotional reactions people may be seeking with their preferred music listening choices. A further 13 items were designed to explore how people interact with music when with friends, and the effects of listening to music both alone and with others, while an additional item specifically focused on the mood effects achieved when listening to self-selected music when sad (Appendix: “Mood Effect”). In addition we also included items relating to the frequency with which people listen to music alone, their use of chat rooms or blogs to talk about music with others, and how much they focus on the lyrics when listening to music.

### Analysis

Statistical analyses were conducted using SPSS 22.0. Since lyrics are known to have an important impact on the effect of music listening (Peynircioğlu and Ali, 2006), an examination of lyrical content was included using Linguistic Inquiry and Word Count (LIWC; Tausczik and Pennebaker, 2010). This software calculates percentages of word categories used in the text including affect words, and use of words relating to specific topics such as motion or death. This method has previously been used to predict coping style (Pennebaker et al., 1997), trauma recovery rates (Pennebaker, 1993), and social integration (Pennebaker and Graybeal, 2001). In particular, given the relationship between self- (Watkins and Teasdale, 2004) and past-focused thinking (Garrido, in press) in the literature with depression, we focused on lyrics relating to the self or the past, and lyrics expressing negative emotions.

## Results

### Preliminary analyses

A principal component analysis (with varimax rotation) was conducted on items relating to group music listening. The items loaded consistently onto two factors (Table 1), one describing positive group interactions around music (36.7% of variance), which was labeled “Positive Group Listening,” and the other describing negative group interactions around music (32.6% of variance) which was labeled “Negative Group Listening.”

**Table 1:** **Factor loading matrix for items relating to group listening**.

**Item code**	**Item**	**Factor 1**	**Factor 2**
MU02	My friends and I like to sit and listen to music and talk about sad things	0.30	**0.83**
MU03	Sometimes when I am with my friends we listen to the same sad songs over and over again	0.31	**0.80**
MU08	Listening to music with my friends sometimes makes me feel depressed	−0.01	**0.84**
MU04	Sometimes when I am with friends we listen to the same happy or inspiring songs over and over again	**0.77**	0.16
MU05	My friends and I like to talk about how the music we listen to is like our own lives	**0.71**	0.40
MU06	My friends and I like to spend a lot of time talking about our favourite band and singers	**0.76**	0.26
MU07	Listening to music with my friends makes me feel really good	**0.84**	−0.00

*Loadings > 0.4 are shown in bold*.

Sub-scales were created using the items that loaded most strongly onto each factor with the Positive and Negative Group Listening subscales returning reliability scores of 0.807 (Cronbach's alpha) and 0.813 respectively. The loading of items in the Negative Group Listening subscale suggests a construct that could be understood to assess “group rumination,” since it involves talking about sad things and listening to sad songs with friends, as well as increased depression from group interactions around music. On the other hand, the Positive Group Listening subscale involves social interactions involving music that result in positive mood outcomes.

A principal component analysis (varimax rotation) was also conducted on items relating to the use of music to either ruminate or reflect. Again, items loaded consistently onto two factors (Table 2), with the exception of one item (Fav02). The first factor was labeled “Ruminating with Music” and explained 25.9% of the variance in responses. The second factor was labeled “Reflecting with Music” and explained 23.8% of the variance.

**Table 2:** **Factor loading matrix for items relating to ruminating and reflecting with music**.

**Item code**	**Scale item**	**Factor 1**	**Factor 2**
SM01	The music I listen to when sad helps me feel my emotions more intensely	**0.52**	0.32
SM03	The music I listen to when sad gives me a reason to be sad	**0.73**	–0.04
Fav01	The kind of music I prefer makes me feel sad	**0.69**	–0.06
Fav02	The kind of music I prefer makes me feel angry	**0.45**	**–0.54**
MU01	Sometimes I can't stop listening to songs that make me think about the past	**0.61**	0.35
MU11	Listening to music reminds me about sad things in my life	**0.78**	–0.06
SM02	The music I listen to when sad helps me to think about my problems and try to sort them out	0.41	**0.67**
Fav03	The kind of music I prefer reminds me of a good period of my life	0.19	**0.46**
MU12	When I am stressed I listen to music to help myself reflect	0.08	**0.80**
MU13	When I am stressed I listen to music to help me gain more positive emotions	−0.16	**0.77**

*Loadings > 0.4 are shown in bold*.

Subscales were created using the items that loaded most strongly on each factor, omitting Fav02. The Ruminating with Music subscale returned a reliability score of 0.710 and the Reflecting with Music subscale returning a score of 0.630 (Cronbach's alphas). The latter scale was omitted from further analysis given the relatively low reliability score. The former scale assesses the use of music to bring back sad memories and intensify negative thoughts and affect.

Pearson's correlation coefficients were calculated in order to explore associations between the scale scores (see Table 3). Ruminating with music was associated with Negative Group Listening as well as with the HUMS Unhealthy subscale and the DASS subscales. It was also negatively correlated with Mood Effect, suggesting that people with high tendencies to ruminate with music were unlikely to experience positive outcomes from listening to their self-selected music when feeling sad. Negative Group listening correlated with the HUMS Unhealthy, and Positive Group listening correlated with the HUMS Healthy (in support of the second hypothesis) In addition, Mood Effect was positively correlated with the HUMS Healthy suggesting that positive mood effects from listening to music are related to the mood regulation strategies being utilized (in support of the third hypothesis). Results also indicated that a reverse relationship existed between age and Positive Group Listening scores

**Table 3:** **Pearson's correlation coefficients for psychometric variables**.

	**Negative group listening**	**Positive group listening**	**Rumination**	**HUMS unhealthy**	**HUMS healthy**	**DASS depression**	**DASS stress**	**DASS anxiety**	**Mood effects**	**Age**
Ruminating with music	*r* = 0.51	*r* = 0.21	*r* = 0.38	*r* = 0.51	*r* = −0.01	*r* = 0.40	*r* = 0.35	*r* = 0.35	*r* = −0.30	*r* = −0.04
	*p* < 0.001	*p* < 0.001	*p* < 0.001	*p* < 0.001	*p* = 0.75	*p* < 0.001	*p* < 0.001	*p* < 0.001	*p* < 0.001	*p* = 0.32
Neg group listening	1	*r* = 0.48	*r* = 0.14	*r* = 0.45	*r* = 0.01	*r* = 0.34	*r* = 0.28	*r* = 0.39	*r* = 0.02	*r* = −0.19
		*p* < 0.001	*p* = 0.001	*p* < 0.001	*p* = 0.83	*p* < 0.001	*p* < 0.001	*p* < 0.001	*p* = 0.64	*p* < 0.001
Pos group listening	*r* = 0.48	1	*r* = 0.06	*r* = 0.13	*r* = 0.39	*r* = 0.02	*r* = 0.11	*r* = 0.17	*r* = 0.15	*r* = −0.32
	*p* < 0.001		*p* = 0.16	*p* = 0.002	*p* < 0.001	*p* = 0.56	*p* 0.006	*p* < 0.001	*p* < 0.001	*p* < 0.001
Rumination	*r* = 0.14	*r* = 0.06	1	*r* = 0.24	*r* = 0.14	*r* = 0.45	*r* = 0.48	*r* = 0.36	*r* = −0.23	*r* = 0.02
	*p* = 0.001	*p* = 0.16		*p* < 0.001	*p* < 0.001	*p* < 0.001	*p* < 0.001	*p* < 0.001	*p* < 0.001	*p* = 0.63
HUMS unhealthy	*r* = 0.45	*r* = 0.13	*r* = 0.24	1	*r* = 0.03	*r* = 0.47	*r* = 0.41	*r* = 0.47	*r* = −0.11	*r* = −0.17
	*p* < 0.001	*p* = 0.002	*p* < 0.001		*p* = 0.40	*p* < 0.001	*p* < 0.001	*p* < 0.001	*p* = 0.005	*p* < 0.001
HUMS healthy	*r* = 0.01	*r* = 0.39	*r* = 0.14	*r* = 0.03	1	*r* = −0.03	*r* = 0.09	*r* = 0.03	r 0.24	*r* = −0.04
	*p* = 0.75	*p* < 0.001	*p* < 0.001	*p* = 0.40		*p* = 0.49	*p* = 0.02	*p* = 0.54	*p* < 0.001	*p* = 0.37
DASS depression	*r* = 0.34	*r* = 0.02	*r* = 0.45	*r* = 0.47	*r* = −0.03	1	*r* = 0.74	*r* = 0.71	*r* = −0.22	*r* = 0.01
	*p* < 0.001	*p* = 0.56	*p* < 0.001	*p* < 0.001	*p* = 0.49		[< 0.001	*p* < 0.001	*p* < 0.001	*p* = 0.76
DASS stress	*r* = 0.28	*r* = 0.11	*r* = 0.48	*r* = 0.41	*r* = 0.09	*r* = 0.74	1	*r* = 0.78	*r* = −0.13	*r* = −0.02
	*p* < 0.001	*p* = 0.006	*p* < 0.001	*p* < 0.001	p = 0.02	*p* < 0.001		*p* < 0.001	*p* = 0.002	*p* = 0.70
DASS anxiety	*r* = 0.39	*r* = 0.17	*r* = 0.36	*r* = 0.47	*r* = 0.03	*r* = 0.71	*r* = 0.78	1	*r* = 0.07	−0.15
	*p* < 0.001	*p* < 0.001	*p* < 0.001	*p* < 0.001	*p* = 0.54	*p* < 0.001	*p* < 0.001		*p* = 0.10	
Mood effects	*r* = 0.02	*r* = 0.15	*r* = −0.23	*r* = −0.11	*r* = 0.24	*r* = −0.22	*r* = −0.13	*r* = −0.07	1	*r* = −0.07
	*p* = 0.64	*p* < 0.001	*p* < 0.001	*p* = 0.005	*p* < 0.001	*p* < 0.001	*p* = 0.002	*p* = 0.10		*p* = 0.07
Age	*r* = −0.19	*r* = −0.32	*r* = 0.02	*r* = −0.17	*r* = −0.04	*r* = 0.01	*r* = −0.02	*r* = −0.15	*r* = −0.07	1
	*p* < 0.001	*p* < 0.001	*p* = 0.63	*p* < 0.001	*p* = 0.37	*p* = 0.76	*p* = 0.69	*p* < 0.001	*p* = 0.07	

### Depression and ruminating with music

As a test of the first hypothesis, participants were grouped according to scores on the DASS Depression subscale based on the cut-off points indicated by the Manual for the Depression Anxiety Stress Scales (Lovibond and Lovibond, 1995). Around 49% of participants in the study reported normal levels of depression, at the time they completed the survey with 51% of participants showing mild to severe levels of depression on this scale.

A MANOVA was conducted with Depression Group as the fixed factor, returning a significant main effect *F*_(5, 608)_ = 24.3, *p* < 0.001, partial-eta squared = 0.1. Follow-up ANOVAs indicated significant differences between groups on Negative Group Listening *F*_(1, 612)_ = 45.9, *p* < 0.001, Cohen's *d* = 0.6; Ruminating with Music *F*_(1, 612)_ = 86.0, *p* < 0.001, Cohen's *d* = 0.8; listening to music alone (Item A1) *F*_(1, 612)_ = 13.5, *p* < 0.001, Cohen's *d* = 0.3; and the use of chat rooms and blogs (Item A2) *F*_(1, 612)_ = 11.8, *p* = 0.001, Cohen's *d* = 0.3, with the Mild to Severe Depression group (*n* = 313) experiencing higher scores than the Normal Depression group (*n* = 301) on all of these variables. In contrast, the Normal Depression group reported significantly higher (more positive) *F*_(1, 612)_ = 25.7, *p* < 0.001 effects of listening to their self-selected music when sad than those with higher levels of depression. These results support the first hypothesis in that people with mild to severe depression were more likely to engage in Negative Group Listening and in ruminating with music generally than those with normal levels of depression. They also suggest that people with normal levels of depression are more likely to benefit from listening to music when sad in support of the third hypothesis.

**Figure 1:**
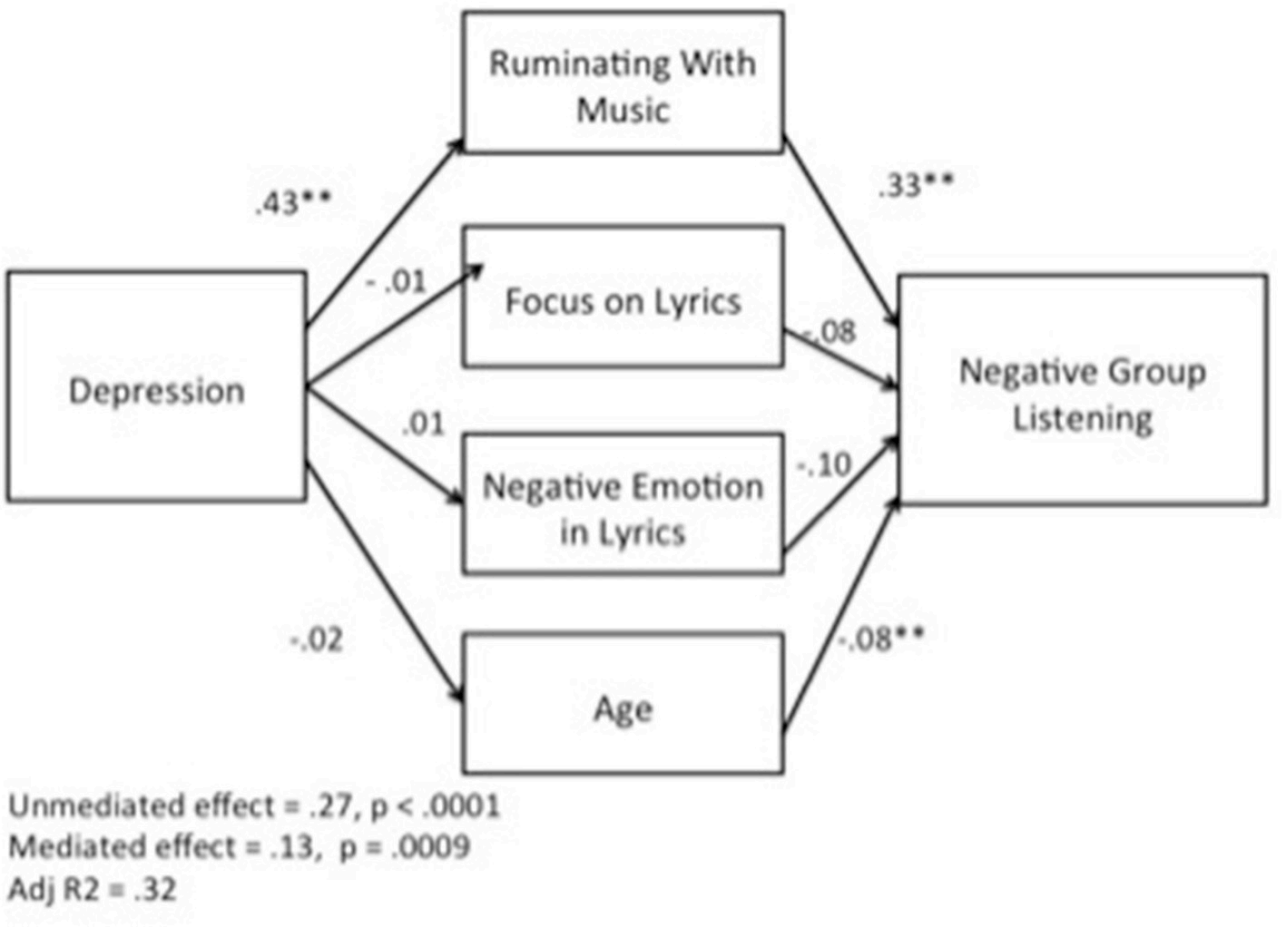
**Indirect effect of depression on Negative Group Listening through Ruminating with Music**. ^**^p < 0.001.

To further explore the relationship between depression and group rumination, a mediation model was tested using bootstrapping methods outlined by Preacher and Hayes (2004) (Figure 1). In this model Ruminating with Music was proposed as a mediator between Depression and Negative Group Listening. Since the lyrical content of the music and the degree to which the listener focuses on lyrics could also be possible influences on the model (Stratton and Zalanowski, 1994), item A3 (Appendix) and the LIWC output for negative emotions in the lyrics were also included in the model. Age was also included based on the suggestion in our previous analysis that age was inversely correlated to positive group listening. Results based on 5,000 bootstrapped samples confirmed the role of Ruminating with music in the relationship between depression and Negative Group Listening, with an unstandardized indirect effect of *B* = 0.14 CI (95%) 0.10 to 0.20 (*p* < 0.05). No other variable in the model had a significant effect. The direct effect of Depression on Negative Group Listening, while still significant [*B* = 0.13, *t*_(359)_ = 3.3, *p* < 0.001), dropped when controlling for Ruminating with Music, indicating partial mediation.

### Adaptive coping styles and music use

A regression analysis was then conducted in order to compare the factors that might be predictive of positive mood outcomes when listening to self-selected sad music. The regression analysis tested the third hypothesis using Mood Effects as the dependent variable and using the enter method with criterion of *F* to enter <= 0.05 and probability of *F* to remove of > = 0.10. As shown in Table 4, the variables that made a statistically significant contribution to the model (at *p* = 0.05) were the HUMS Healthy subscale, and Negative Group listening, while Ruminating with music,), Depression, Anxiety, rumination, and the use of “I” words in the lyrics were inversely predictive of positive mood effects. The model explained 22% of the model variance (adjusted *R*^2^). This supported the third hypothesis, demonstrating that adaptive coping styles were predictive of experiencing positive mood outcomes from music listening when sad.

**Table 4:** **Regression model of Mood Effects of Self-Selected Music when Sad**.

**Dependent**	***m***	***SD***	**B**	**Std. error**	**Correlation with mood effects**	**β**	***t***	**Sig**.	**VIF**
(Constant)			2.0	0.3			7.0	<0.001	
HUMS healthy	14.9	3.3	0.1	0.0	0.24	0.2	5.0	<0.001	1.3
Negative group listening	−3.9	4.6	0.1	0.0	0.02	0.2	4.0	<0.001	2.0
HUMS unhealthy	10.2	6.3	0.0	0.0	−0.11	0.0	0.4	0.69	1.7
Positive group listening	2.3	5.9	0.0	0.0	0.15	0.0	0.3	0.80	1.7
Reflectiveness	3.4	0.8	−0.0	0.1	−0.01	−0.0	−0.5	0.62	1.1
Ruminating with music	0.1	6.1	−0.1	0.0	−0.30	−0.3	−7.4	<0.001	1.8
DASS depression	6.1	5.6	0.0	0.0	−0.22	−0.2	−4.0	<0.001	2.8
Rumination	3.6	0.8	−0.2	0.1	−0.23	−0.2	−4.5	0.001	1.5
“I” words	9.6	5.1	0.0	0.0	−0.11	−0.1	−3.0	0.003	1.3
DASS anxiety	5.1	4.8	0.0	0.0	−0.07	0.1	2.3	0.022	3.1
DASS stress	6.2	5.0	0.0	0.0	−0.13	0.0	0.3	0.77	3.4
Past words	3.1	2.6	−0.0	0.0	−0.02	−0.0	−0.6	0.56	1.1
Neg Emo words	2.7	2.3	−0.0	0.0	−0.05	−0.0	−0.6	0.55	1.0

A further regression analysis was then conducted in order to test the fourth hypothesis that people with adaptive coping styles would use music in uplifting or positive ways in group settings. Positive Group Listening was the dependent variable, and the enter method was used with criterion of *F* to enter < = 0.05 and probability of *F* to remove of >= 0.10. Table 5 demonstrates that statistically significant contributions to the model were Ruminating with Music, DASS Anxiety and the HUMS Healthy subscale. Predictors with an inverse relationship to the dependent variable were Rumination and DASS Depression. The strong result regarding the HUMS Healthy subscale supports the fourth hypothesis, in that adaptive coping styles were seen to be predictive of Positive Group Listening. Interestingly, while Depression was inversely predictive of Positive Group Listening, Anxiety was a positive predictor, suggesting that people with Anxiety may benefit more from group interactions with music as opposed to people with Depression.

**Table 5:** **Regression model of Positive Group Listening (PGL) with Background Variables**.

**Dependent**	***M***	***SD***	**B**	**Std. error**	**Correlation with PGL**	**β**	***t***	**Sig**.	**VIF**
(Constant)			−5.8	1.5			−3.5	<0.001	
Ruminating with music	0.1	6.1	0.2	0.0	0.21	0.2	5.6	<0.001	1.5
DASS anxiety	5.1	5.8	0.3	0.1	0.17	0.3	4.5	<0.001	3.0
DASS depression	6.1	5.6	−0.2	0.1	0.02	−0.2	−3.1	0.002	2.7
DASS stress	7.2	4.8	−0.6	0.1	0.11	−0.0	−0.7	0.469	3.3
HUMS unhealthy	10.2	6.3	−0.0	0.0	0.13	−0.0	−0.4	0.685	1.7
HUMS healthy	15.0	3.3	0.7	0.1	0.39	0.4	10.6	<0.001	1.1
Rumination	3.6	0.8	−0.7	0.3	0.05	−0.1	−2.1	0.037	1.5
Reflectiveness	3.4	0.8	0.1	0.3	0.10	0.0	0.2	0.819	1.1
“I” words	9.6	5.1	−0.0	0.0	0.02	−0.0	−0.2	0.859	1.0
Past words	3.1	2.6	−0.1	0.1	−0.02	−0.0	−1.2	0.217	1.0
Neg Emo words	2.7	2.3	0.0	0.1	0.01	0.0	0.1	0.899	1.0

## Discussion

The results suggested two distinct patterns of behavior involving group music listening: (i) listening to sad music and talking about sad things—a form of group rumination—and (2) listening to inspiring music and discussing music and life more generally. Items relating to the former construct aligned with the item indicating feeling more depressed after listening to music with friends, while positive interactions with music loaded onto the same factor as the item indicating that listening to music with friends felt good. This suggests that both types of behavior have distinct outcomes. Correlation analyses also confirmed that the two types of group interactions (positive and negative) were associated with either healthy or unhealthy ways of using music respectively.

Similarly, items relating to ruminating with music loaded clearly together as did items relating more closely to Trapnell and Campbell's (1999) dimension of reflection, suggesting again, that behaviors relating to music use fall into distinct patterns, reflecting either healthy or unhealthy thought processes. Negative group interactions around music also tended to occur more often in younger participants, likely reflecting the relative importance of both music and social relationships to younger people (McFerran et al., 2014).

The results also reveal important information about the use of music by people with depression. People with depression in our sample reported listening to music alone more than people with normal levels of depression. This is not surprising given that social withdrawal is generally associated with anxiety and depression (Rubin and Coplan, 2010). They were also more likely to use online chat rooms and blogs relating to music than other participants, in harmony with studies suggesting increased media use in people with depression (Block et al., 2014). However, negative group interactions around music were also higher in the depressed group than in those with no depression, and were higher in those who tended to ruminate with music generally. Thus, while depressed individuals may often seek solitude, when social interactions around music occur these may often be ruminative in nature and result in negative outcomes. The fact that the relationship between Depression and Negative Group listening was mediated by Ruminating with Music suggests that these negative outcomes are influenced by a tendency to use music for ruminative purposes.

Interestingly, regression analyses suggested that while this is so for people with depression, for people with anxiety, group interactions about music may tend to be more positive, even where such individuals have a tendency to ruminate with music. Such positive outcomes were strongly predicted by scores on the HUMS Healthy subscale. This scale assesses behaviors such as using music to connect with other people and to relax. Thus, it may be that people with anxiety who tend to use music ruminatively when alone may actually benefit from the social connections that music gives them.

Healthy uses of music were also associated with positive effects from listening to self-selected music when sad other than in group contexts. Another predictor of positive effects was Negative Group listening. While the inclusion of group rumination as a predictor of positive mood effects was surprising, it perhaps suggests that people who find group interactions around music to have negative effects may find listening to music alone more beneficial if healthy listening strategies are being employed. Conversely, listening alone to self-focused music in the context of ruminative behavior appears to be inversely related to positive mood outcomes. Thus, the effect of group music listening appears to be very much related to the coping style being used, rather than to whether people listen to music alone or in groups.

## Conclusions

The present study has demonstrated that the outcomes of both solitary and group music listening are related to the thinking patterns and coping styles being employed in both settings. People with generally maladaptive coping styles tend to report negative outcomes from both listening to music alone and from group interactions around music. It appears that this occurs because such people are more likely to engage in ruminating with music - using music both to intensify negative affect and to focus on negative thoughts and memories.

Young people with depression may be particularly vulnerable to the impacts of group rumination. While young people with tendencies to depression who are a part of social groups may be perceived as receiving valuable social support, our results here suggest that the positive impacts of such group interactions are reliant on the types of processes occurring in the group. Thus it seems that strong social links—something that usually protects against depression—can, in ruminators, lead to an increased focus on negative emotions resulting in increased depression in at risk individuals.

Music can facilitate the sharing of negative emotions. Where this occurs within a group of distressed individuals, it seems that group rumination by listening to depressing music and focusing on negative thoughts and events, can tend to amplify and nourish the maladaptive thought patterns implicit in the group, with potentially dangerous results (Garrido, 2017). The actual outcomes from this kind of sharing of emotions around music may be dependent on the dominant affective responses and thinking patterns of the individuals in the group. While this was not directly measured in this study, the research presented here suggests that neither certain genres of music themselves nor the sub-cultures associated with them, can be blamed for situations in which music appears to have played a role in youth suicides. However, susceptible individuals with a predilection for rumination may be most likely to suffer negative outcomes from group rumination, with social feedback deepening and exacerbating negative thoughts and feelings. Where group interactions provide social support or opportunities for cognitive reframing and processing of emotions such as where such group discussions are carefully facilitated by a therapist, the effects are more likely to be positive (Zhou et al., 2015).

Thus, these findings have important implications for addressing depression in young people and in understanding social interactions around music in vulnerable individuals. The study is limited by its basis on cross-sectional data. Thus it cannot be assumed that the same results would be found in a longitudinal study or that the direction of the relationship between depression and group rumination is clearly established. While the current study tested the idea that people with depression were more likely to engage in group rumination and to experience negative mood outcomes from such group interactions, future research should aim to assess causal relationships more clearly in experimental settings. Furthermore, despite the fact that some of the group reported low scores in depression, participants were recruited through mental health websites and discussion boards related to depression. Many participants who did not report current depression may not be completely removed from behavior common in depression. Thus, further study will also be required to clarify how the dynamics of group interactions influence mood outcomes and whether the impact of group rumination is likely to be more or less dangerous than solitary forms of rumination, comparing people with depression to a healthy control group.

## Ethics statement

The study received ethics approval from both the Human Research Committee of the University of Melbourne, Australia, and the Department of Music Human Research Ethics Committee at Durham University, UK. The research was conducted by means of an online survey. The opening page of the survey outlined the purpose and details of the study and participants were advised that if they clicked “Next” to continue with the survey they were consenting to participate in the study under those terms. Since the study involved people with depression participants were questioned at the end of the survey as to whether participation in the study had made them feel worse. An answer of “yes” from a participant triggered an automatic email alert to the researchers who were able to follow up with advice as to appropriate local sources for mental health support.

## Author contributions

SG was the primary author involved in research concept, design of work, data acquisition, interpretation of data and drafting of the manuscript. TE provided substantial contribution to the acquisition and interpretation of data and drafting of the manuscript. KM provided substantial contribution to the design of the work, interpretation of the data and revising of the manuscript.

## Funding

This work was partially supported by an Australian Research Council Discovery Project DP110102483 grant to the third author.

### Conflict of interest statement

The authors declare that the research was conducted in the absence of any commercial or financial relationships that could be construed as a potential conflict of interest.
