# Supplementary material for: Group Rumination: Social Interactions Around Music in People with Depression
Source: Front Psychol. 2017 Apr 4;8:490. doi: 10.3389/fpsyg.2017.00490 (PMC5378782; doi:10.3389/fpsyg.2017.00490)
Supplement: Supplementary file 1 [file DataSheet1.docx]

**Appendix – Survey Items**

| Item Code | Item Text (rated on a scale of -3 = Strongly disagree to 3 = Strongly Agree) |
| --- | --- |
| SM01 | The music I listen to when sad helps me feel my emotions more intensely |
| SM02 | The music I listen to when sad helps me to think about my problems and try to sort them out |
| SM03 | The music I listen to when sad gives me a reason to be sad |
| Fav01 | The kind of music I prefer makes me feel sad |
| Fav02 | The kind of music I prefer makes me feel angry |
| Fav03 | The kind of music I prefer reminds me of a good period of my life |
| MU01 | Sometimes I can’t stop listening to songs that make me think about the past |
| MU02 | My friends and I like to sit and listen to music and talk about sad things |
| MU03 | Sometimes when I am with my friends we listen to the same sad songs over and over again |
| MU04 | Sometimes when I am with friends we listen to the same happy or inspiring songs over and over again |
| MU05 | My friends and I like to talk about how the music we listen to is like our own lives |
| MU06 | My friends and I like to spend a lot of time talking about our favourite band and singers |
| MU07 | Listening to music with my friends makes me feel really good |
| MU08 | Listening to music with my friends sometimes makes me feel depressed |
| MU09 | Listening to music alone makes me feel really good |
| MU10 | Listening to music alone sometimes makes me feel depressed |
| MU11 | Listening to music reminds me about sad things in my life which makes me feel sadder than before |
| MU12 | When I am stressed I listen to music to help myself reflect |
| MU13 | When I am stressed I listen to music to help me gain more positive emotions |
| MoodEffects | After listening to your self-selected music when feeling sad, do you usually feel… (-3 = a lot worse, 3 = a lot better) |
| A1 | How often are you alone when listening to music? (0 = Never, 4 = Always) |
| A2 | Do you ever use chat rooms or read blogs about your preferred music style? (0 = Never, 6= Every day) |
| A3 | How much do you tend to focus on the lyrics when listening to music (0 = Not at all, 4 = A lot) |
